# A Smartphone App to Promote an Active Lifestyle in Lower-Educated Working Young Adults: Development, Usability, Acceptability, and Feasibility Study

**DOI:** 10.2196/mhealth.8287

**Published:** 2018-02-20

**Authors:** Dorien Simons, Ilse De Bourdeaudhuij, Peter Clarys, Katrien De Cocker, Corneel Vandelanotte, Benedicte Deforche

**Affiliations:** ^1^ Health Promotion and Education Unit Department of Public Health Ghent University Ghent Belgium; ^2^ Physical Activity, Nutrition and Health Research Unit Faculty of Physical Education and Physical Therapy Vrije Universiteit Brussel Brussels Belgium; ^3^ Department of Movement and Sport Sciences Faculty of Medicine and Health Sciences Ghent University Ghent Belgium; ^4^ Physically Active Lifestyles Research Group Institute for Resilient Regions University of Southern Queensland Springfield Central Australia; ^5^ Physical Activity Research Group School for Health, Medical and Applied Science Central Queensland University Rockhampton Australia

**Keywords:** mHealth, young adult, mobile applications, physical activity, active transport, health promotion

## Abstract

**Background:**

Physical activity (PA) levels are problematic in lower-educated working young adults (18-26 years). To promote PA, smartphone apps have great potential, but there is no evidence for their effectiveness in this population. To increase the likelihood that a newly developed app will be effective, formative research and user testing are required.

**Objective:**

The aim of this study was to describe the development, usability, acceptability, and feasibility of a new theory- and evidence-based smartphone app to promote an active lifestyle in lower-educated working young adults.

**Methods:**

The new app was developed by applying 4 steps. First, determinants important to promote an active lifestyle in this population were selected. Second, evidence-based behavior change techniques were selected to convert the determinants into practical applications. Third, a new smartphone app was developed. Fourth, volunteers (n=11, both lower and higher educated) tested the app on usability, and lower-educated working young adults (n=16) tested its acceptability and feasibility via (think aloud) interviews, a questionnaire, and Google Analytics. The app was accordingly adapted for the final version.

**Results:**

A new Android app, *Active Coach*, was developed that focused on knowledge, attitude, social support, and self-efficacy (based on outcomes from step 1), and that applied self-regulation techniques (based on outcomes from step 2). The app consists of a 9-week program with personal goals, practical tips, and scientific facts to encourage an active lifestyle. To ensure all-day and automatic self-monitoring of the activity behavior, the Active Coach app works in combination with a wearable activity tracker, the Fitbit Charge. Issues detected by the usability test (eg, text errors, wrong messages) were all fixed. The acceptability and feasibility test showed that participants found the app clear, understandable, and motivating, although some aspects needed to be more personal.

**Conclusions:**

By applying a stepwise, user-centered approach that regularly consulted the target group, the new app is adapted to their specific needs and preferences. The Active Coach app was overall positively evaluated by the lower-educated working young adults at the end of the development process.

## Introduction

Emerging adulthood is a period ranging from the late teens through the twenties and comprises various turning points in life such as changes in education, employment, or place of residence [1,2]. These changes have shown to be associated with a decrease in physical activity (PA) and active transport (AT) levels [3-6], making young adults (18-26 years) an important target group for the promotion of an active lifestyle. Additionally, young adults’ PA and AT choices are likely to remain stable over time and provide long-term health benefits in adulthood [7,8]. In Belgium, approximately 50% of 15-24 year olds does not reach the recommended 30 min of moderate PA a day [9], which increases their all-cause mortality risk with 11.4% [10]. AT represents an opportunity to include PA into young adults’ busy daily life [11]. Young adults who started working around the age of 18 years and who did not complete higher education (college or university) have an even higher risk for inactivity because of their lower educational attainment. Among adults of all ages, lower levels of education have been associated with lower levels of general PA [2,12], less AT [13,14], and higher levels of overweight and obesity, and prevalence of common chronic diseases [15]. As such, there is a clear need to promote an active lifestyle in lower-educated working young adults.

Recent technologies such as smartphones, health and fitness apps, and consumer wearable activity trackers have great potential as tools for assessing and promoting PA in all age groups [16-20]. Smartphone apps can measure PA and AT and provide feedback in real time; provide interactive, individualized, and automatically generated content; and deliver materials on a device (ie, smartphone) that is already carried by the individual [21]. In addition, consumer wearable activity trackers are a popular and growing market for monitoring PA and can be used in combination with smartphones [20]. Smartphones are gaining popularity worldwide, and they are most popular among young adults. In the United States [22] and Belgium [23], respectively, 85% and 80% of young adults own a smartphone. Young adults and lower socioeconomic subgroups in high-income countries tend to use mobile phones more compared with other age groups and high socioeconomic subgroups [22,24-26]. Due to their potential and popularity, smartphone apps might be a good tool to promote an active lifestyle in lower-educated working young adults.

Many PA apps are already available through app stores such as Apple App Store and Google Play Store. However, most of these apps are not developed in collaboration with health professionals or academics, do not incorporate theoretical content, and have a relative absence of evidence-based behavior change techniques (BCTs) [27-32]. Additionally, the existing PA apps might not be appropriate for certain target groups such as lower-educated working young adults, as they are not specifically adapted to their lifestyle and cognitive capacities. Therefore, developing a new theory- and evidence-based PA app tailored to the needs of this target group is necessary.

Developing a new theory- and evidence-based smartphone app requires conceptualization (reviewing evidence, understanding of the needs and perspectives of the intended users, deciding on the theoretical basis, planning the developmental process), formative evaluation, and pretesting the acceptability (is the target group willing to receive the strategies?) and feasibility (is it realistic to consider implementing the proposed strategies?), before evaluating its effectiveness [33-38].

Only a few mobile health (mHealth) studies have described these developmental steps in detail [34]. It is important to present this process to help others in developing effective tools to improve health [33]. Therefore, the aim of this study was to describe the development, acceptability, and feasibility of a new smartphone app (Active Coach) to promote an active lifestyle in lower-educated working young adults.

## Methods

### Approach

A stepwise approach, consisting of 4 steps, based on the Intervention Mapping Approach and the developmental steps for mHealth interventions, was used in the development of the new app [34,39]. In step 1, determinants important to promote an active lifestyle among low-educated working young adults were selected. In step 2, evidence-based BCTs [40] were selected to convert the determinants into practical applications. In step 3, a new smartphone app called “Active Coach” was developed. In step 4, the Active Coach app was tested on errors, acceptability, and feasibility and accordingly adapted for the final version. In this chapter, only the methods of the 4 steps will be described. The results (including the content of the app) will be described next in the *Results* section. This study was approved by the ethics committee of the university hospital of Ghent University (B670201525362) and by the ethics committee of the Vrije Universiteit Brussel (BUN 143201112745).

### Step 1: Selecting Determinants

To develop an evidence- and theory-based app, determinants important to promote an active lifestyle in lower-educated working young adults need to be selected. The selection was based on the existing literature, previous studies from our research group, a theoretical health behavior change model, and an exploratory qualitative study among lower-educated working young adults (see Results step 1 [4,41-57]).

In the qualitative study, focus groups were conducted among lower-educated working young adults to assess determinants of an active lifestyle (PA and AT). In addition, opinions about mobile technologies (eg, smartphones, tablets, apps, fitness trackers) and valuable features of PA apps and their use to promote an active lifestyle were explored. Eligible participants had to be employed, aged between 18 and 26 years, and without a university or college degree (lower educated). Participants (n=34, mean age: 24.0 [SD 3.0] years, 59% [20/34] men, mean years of employment: 3.0 [SD 2.0]) were recruited throughout Flanders (northern part of Belgium). Snowball and convenience sampling, two nonprobability approaches often used in qualitative research [58], were used to recruit participants via the personal network of researchers and assistants and via social media. Five focus groups (6-10 participants per group) were conducted at places that were most convenient for the participants. Focus groups were conducted until saturation, which is the point at which all questions have been thoroughly explored in detail and no new concepts or themes emerge in subsequent sessions [59]. All focus groups were held in Dutch and lasted approximately 60 min. A focus group protocol and a semistructured discussion guide (Table 1) were developed consistent with the recommended focus group methodology [60]. The guide consisted of several questions, including an introduction question, a transition question, key questions, and an ending question. For some questions, participants were asked to write down an answer. This method allows participants to think and reflect about a question before starting a group discussion and not to copy other participants’ answers or opinions. Before the discussion started, the participants provided informed consent and completed a brief questionnaire obtaining sociodemographic information. The discussions were led by a moderator. Notes were taken by an observer. With permission of the participants, all conversations were audiotaped for transcription. The focus group interviews were transcribed verbatim, and the texts were incorporated into a qualitative processing program (NVivo 9 qualitative software, QRS International). The data were analyzed based on grounded theory. Grounded theory is a method of analyzing qualitative data without preconceived theories and is characterized by intensively analyzing data, often sentence by sentence or phrase by phrase [61]. During the transcription of the conversations, we developed codes according to the responses and the themes that arose frequently and were relevant to the aim of the study. Data obtained by the questionnaire were entered into SPSS (version 23.0) to calculate descriptive statistics.

### Step 2: Selecting Behavior Change Techniques

BCTs were used to translate the selected determinants into practical applications that will be used in the new app [40]. BCTs are the active component of an intervention designed to change behavior [62]. Michie et al [40] developed a BCT taxonomy of 93 hierarchically clustered techniques. BCTs were selected based on their previously demonstrated effectiveness and on the focus group results (see Results step 2 [25,39,40,63-70]).

### Step 3: Developing the App

On the basis of the translation of the BCTs into practical applications and on the basis of the results from the focus groups, a new app was developed in cooperation with a commercial mobile app development company (Cucumber Apps). It is a native Android app (only developed for the Android operating system), which means that it is completely compatible with the smartphone’s native features and hardware (eg, accelerometer, camera, GPS) and ensures the best user experience [71]. In 2015, Android was the operating system with the highest penetration rate in the smartphone market worldwide (80% Android vs 19% IOS) [72] and among 15- to 35-year-olds in Belgium (57% Android vs 37% IOS) [23]. Moreover, developing an app for iPhone (IOS) would be too expensive and too time-consuming.

The content of the app was developed to incorporate an autonomy-supportive communication style, based on the self-determination theory [73]. Self-determination theory suggests that the content of goals (ie, intrinsic vs extrinsic) and the way goal contents are communicated (ie, autonomy-supportive vs controlling) explain variance in people’s motivation and performance [74]. Autonomy-supportive includes a more motivating language (can, may, want) instead of a controlling language (should, have to). The first version of the intervention content was read and evaluated by junior and senior researchers in the field of public health (n=19, mean age: 26.2 [SD 5.1] years, 26% [5/19] men). They were consulted because of their experience and knowledge regarding public health interventions. After adapting the first version, interviews were held with lower-educated working young adults (n=10, mean age: 23.0 [SD 2.0] years, 50% [5/10] men) to check the usefulness, applicability, and understandability of the content. Participants were recruited using convenience sampling, and the interviews were audiotaped and transcribed verbatim. Analyses were conducted as described in step 1.

### Step 4: Testing the App on Usability, Acceptability, and Feasibility

First, usability of the app was tested by a small group of volunteers (n=11, mean age: 28 [SD 10] years, 55% [6/11] men, both lower and higher educated) who checked the app for faults and errors (also known as “bugs”). Nielsen et al [75] showed that conducting usability testing with only 5 participants will reveal 85% of usability problems. These volunteers (all different from the focus group participants recruited in step 1) needed to own an Android smartphone and were recruited via the personal network of researchers and colleagues in 2016 (convenience sampling). They installed the app and used it for approximately 5 weeks. All problems mentioned by the volunteers were collected by the researchers via an issue list and passed on to the developers. Accordingly, the app was adapted.

**Figure 1:**
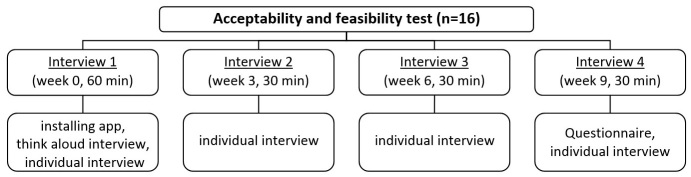
Flowchart of the acceptability and feasibility test (Step 4).

Next, the adapted version of the app was tested on acceptability and feasibility by lower-educated working young adults. A contact list of the previously conducted focus groups (see step 1) was used as a basis to recruit participants via snowball and convenience sampling. In addition, participants were recruited via a social employment business with several projects for lower-educated people (VZW Ateljee, Ghent). A total of 4 participants of the acceptability and feasibility test had participated in the previously conducted focus groups; all other participants were new. Participants (n=16, mean age: 24.4 [SD 2.3] years, 63% [10/16] men, mean years of employment: 3.7 [SD 2.3] years) had to possess an Android smartphone. During the acceptability and feasibility test, 4 interviews were conducted with each of the participants during a 9-week period (1 interview every 3 weeks; see Figure 1).

**Table 1:** Focus group semistructured discussion guide. PA: physical activity.

Question type	Purpose	Question
Intro	To begin discussion of topic	Write down 5 reasons why you are or would like to be physically active.
Transition	To move toward the key questions	Now we are going to discuss your answers.
		How much or how often do you think you need to be physically active to stay healthy?
		Do you think you are physically active enough to stay healthy?
		Is your amount or level of PA changed since you started working? Why?
Key	To obtain insight about determinants of PA	Would you like to be more physically active?
		Write down 3 activities that involve PA and that you would like to do, but that you do not do for one reason or another.
		Write down 5 reasons why you would not be physically active enough or difficulties you have to be regularly physically active.
		Now we are going to discuss your answers.
		Which solutions may help you to overcome these barriers that you just summed up?
		Have you ever tried to be more physically active? How?
Transition	To move toward the key questions on mobile technologies and PA apps	Do you have a smartphone? How often do you use your smartphone?
		Why do you use your smartphone? (short message service, calls, apps, internet, etc)
		Do you use PA apps, such as Runkeeper, runtastic? Why (not)? What do you think of those apps?
		Does anyone use a fitness tracker, a device or bracelet that tracks your activity? Do you know it? What do you think of it?
Key	To obtain insight about mobile technologies and PA apps	This is an example of a smartphone app (Stappenteller) that tracks your daily steps. What do you think of it? What do you think of the design of this app? Do you think this app is clear, do you understand everything? This app can only work when you have your smartphone with you. Do you always have your smartphone with you? Yes or No? Why (not)? Is it feasible for you to always carry your smartphone with you? Would you like to use this app? Why (not)? Could this app help you to become more active? Why (not)?
		Do you have a computer, laptop or tablet at home? (How often) do you use it? Do you have Internet access on those devices?
		Who has a Facebook account? How often do you use it? Do you also use other social networks such as Twitter, Instagram, LinkedIn, Google Plus...?
		Would you like to get information about the importance of PA and how you can increase your PA? Why (not)? What information would you like? Would you prefer reading that information on your computer (website) or on your smartphone (app)?
		Here is an example of a page (Can be a website or an app) on which information and tips about PA are shown. On this page a few questions are asked so that the information and tips can be personalized. A PA goal is also set. Would you fill in those questions to receive personalized information? What do you think about the question to fill in your step count? (would you fill it in? Would you cheat? Does it need to be automatic?) What do you think about the goal that is set? Would you rather choose your own goal? What do you think of the information displayed here? Do you think this page should be linked to Facebook or another online social network? Would you like to share your results or progress with others? Why (not)? If this page was a website, how much would you use it? Why? How long would you use the page and the given information? Why?
Ending	To bring closure to the discussion	Does anyone have suggestions or additions?

During the first interview (week 0), participants were informed about the purpose of the study. They were asked to sign an informed consent form and to complete a questionnaire about sociodemographics. A think-aloud interview was conducted during the download, install, registration, and first use of the app. In the think-aloud interview, participants were asked to use the app and say out loud any thoughts that came to mind. This is particularly useful as people give their immediate reactions to every element of the app, and it allows researchers to observe how it was used [76]. Afterward, a brief semi-structured interview (Table 2) was conducted to discuss issues that came up during the think-aloud interview and to ask additional questions. During the second (week 3) and the third (week 6) interview, the same semistructured interview was conducted to obtain intermediate information about participants’ experiences with the app (Table 2). During the fourth interview (week 9), a paper-and-pencil questionnaire, based on existing questionnaires assessing acceptability and feasibility, was used to evaluate specific elements of the app [77,78]. Moreover, 4 questions on a 5-point scale from 1 (strongly disagree) to 5 (strongly agree) were used to assess general opinions about the app (ie, clear, fun, user-friendly, attractive), opinions about the tips and facts (ie, interesting, motivating, boring), and opinions about the goals received (ie, motivating, useful, tried to achieve it). After completing the questionnaire, a semistructured interview was held to discuss the answers of the questionnaire in more detail (Table 2). All interviews were held in Dutch and were audiotaped for transcription with permission of the participant. Analyses were conducted as described in step 1. In addition, Google Analytics [79] was used to obtain app usage statistics and evaluate how participants used the Active Coach app. Google Analytics offers free tools to measure website and app data to gain usage insights.

**Table 2:** Semistructured discussion guide.

Questions during interview 1, 2, 3, and 4	Additional instructions
So far, what do you like or dislike about the Active Coach app?	What did you find good or not good?
	Why do you think that was good or not good?
	Can you tell a bit more about that?
Are there certain parts of the app that you find confusing or that you do not understand?	Which parts are confusing?
	Can you tell me more about that?
	Why do you think that?
	How could this be improved?
What change(s) would you recommend to improve the app?	Design, color, font, content, ease of use
	Why do you think that would improve the app?
	Can you tell me more about that change?
Are there certain parts of Active Coach app that definitely should stay the same?	What are they?
	Can you tell me a bit more about that?
	Why do you think that?
What do you think of the use of the Fitbit Charge?	Why do you think that?
	Can you tell me a bit more about that?
Are there any problems or difficulties while using the Active Coach app or the Fitbit?	Which one?
	Can you tell me a little more about that?
What do you think of the daily and weekly goals?	Do you manage to achieve your goal?
	How useful do you find these goals?
	Have these goals helped you to be more active?
	What do you like or dislike about the goals?
	Can you tell me a little more about that?
What do you think of the tips you get each Monday and Friday?	Have you read the tips and facts?
	Do you sometimes reread the tips and facts?
What do you think of the facts you get each Wednesday?	How useful do you find these tips and facts?
	Do these tips or facts help you to be more active?
	What do you think of the amount of tips and facts?
	Would you like to get more or less?
	What do you like or dislike about the tips and facts?
	Can you tell me a little more about that?
Do you use the app regularly?	Do you use the app more, the same, or less than in the beginning? Why?
What makes you continue to use the app?	Would you continue to use the app if you were not participating in a study? Why (not)?
What would be the main reason for you to stop using the app?	Why?
	How could we change this?

## Results

### Step 1: Selecting Determinants

From the existing literature, self-efficacy has been shown to be one of the most important determinants of PA [41-43] and AT [44-46] among (young) adults. Social support was positively associated with PA and AT in adolescents [42] and young adults [4,47]. A review also showed that social support and having a companion for PA were positively associated with different types of PA, including AT [57]. Attitude has been an inconsistent determinant in the literature [42]. However, important perceived benefits and barriers of PA and AT have been mentioned in recent studies. Health benefits, recreation (releasing tension), social contact, and body image have shown to be important benefits for young adults’ PA participation [52,53]. Important benefits of AT among young adults are low costs, autonomy and flexibility, and a short travel time in urban areas [47,49]. Barriers of PA are lack of time, lack of motivation, and lack of money [50,51]. Barriers of AT are bad weather, practicality (eg, how to deal with luggage), comfort (eg, sweating), and lack of facilities such as bicycle parking or showers and changing rooms at work [44,47,48]. Although knowledge might not be sufficient to change behavior, it is a necessary prerequisite to an individual’s positive motivation to engage in more PA [56]. It has been shown that knowledge of PA guidelines in Irish and English adults is very low and that lower education is associated with not knowing the guidelines [54,55].

In the focus groups conducted in this study, these results were confirmed by the target group. For example, lower-educated working young adults mentioned similar perceived benefits and barriers of PA and AT as found in the existing literature. They also indicated to have a lack of knowledge regarding an active lifestyle, and they were very interested in information and advice. Other results of the conducted focus groups (regarding mobile technologies, valuable app features, and app use to promote an active lifestyle) are discussed in steps 2 and 3 of the results section.

As a result, following determinants of an active lifestyle in lower-educated working young adults were selected: knowledge, attitude (perceived benefits and perceived barriers), social support, and self-efficacy. Therefore, the attitude–social influence–self-efficacy (ASE) model [80] was used as a base to develop an app for the promotion of an active lifestyle. The ASE model is a theoretical model that describes the processes wherein health behaviors, such as an active lifestyle, are shaped. It states that an active lifestyle is defined by intention to act, whereas intention, in turn, is determined by attitudes (ie benefits and barriers), social influences, self-efficacy, and the knowledge and skills needed to achieve an active lifestyle [80]. The ASE model has been used in the development of previous health interventions [81-84].

### Step 2: Selecting Behavior Change Techniques

BCTs were used to translate the selected determinants into practical applications [40] (Table 3). Multiple self-regulation techniques were selected (self-monitoring, goal-setting, feedback on behavior, review of behavior goals, instruction on how to perform the behavior) as it has been shown that these techniques are important to target the selected determinants to increase PA in interventions [63-66]. Moreover, both in qualitative and quantitative studies, young adults rated self-regulation techniques (especially self-monitoring and goal-setting) as most valuable features to increase self-efficacy among health behavior apps [25,85]. In addition, participants of the focus groups conducted for this study also mentioned self-regulation techniques (self-monitoring, goal-setting, and instruction on how to perform the behavior) as valuable app features. Furthermore, to encourage self-efficacy regarding the (re)use of the app, “prompts/cues” was selected as a BCT. Providing prompts or cues (notifications in an app) has had a positive effect on reuse of intervention websites among adults, adolescents, and children, particularly those with low socioeconomic status [68-70]. A qualitative study among young adults also found that relevant and timely (but not too frequent) alerts and reminders are valuable features of health behavior apps [25]. Furthermore, participants of the current focus groups mentioned push notifications as necessary to not forget an app.

The BCTs “instruction on how to perform the behavior” and “information about health consequences” were selected to increase knowledge and to encourage a positive attitude toward PA and AT. A qualitative study among young adults showed that providing feedback and advice to guide people about how they can change behavior was evaluated as a valuable app feature [25]. Participants of the focus groups conducted in this study also indicated to be interested in information and advice regarding an active lifestyle:

I would find it interesting to know how active I need to be to be healthy. But I need encouragement. And people need guidelines and ideas on how to be active.

Finally, the BCT “enhancing network linkages” was selected. This BCT focuses more indirectly on social support by advising on mobilizing and maintaining social networks (eg, tips on being active together) [39,67]. Although social support is positively associated with an active lifestyle, it has been found that smartphone users do not like apps that link and share (health) information with social network sites [25,67]. In the conducted focus groups, Facebook was indicated as the most popular social network. Participants used it daily for communication or games, but they did not want to post or share health-related information on Facebook via an app.

### Step 3: Developing the App

The new native Android app, Active Coach, aims to promote an active lifestyle in lower-educated working young adults via a 9-week program. There is no consensus on the optimal duration of app programs to ensure user engagement [18]. However, in a recent review on app interventions to improve health behavior (ie, PA), intervention durations longer than 8 weeks tended to be effective [18]. Nevertheless, very lengthy app programs might not be useful, as app use often declines rapidly because people lack commitment and use apps in a transient, casual way [25,86].

Users of the Active Coach can choose how they want to make their lifestyle more active, through general PA or through AT. Participants of the focus groups conducted in this study agreed that a smartphone app would be used more and more suitable to promote an active lifestyle than a website:

...a smartphone is way easier than a computer. You always have it by your side and you don’t have to wait until it’s ready to be used.

Although focus group participants used their smartphone on a daily basis, many of them indicated that they were not allowed to carry their smartphone with them during working hours. However, they clearly preferred automatic tracking of their activity behavior:

I don’t want to enter anything myself. It takes time and you could cheat and maybe you do it ones, or twice, but not more.

Therefore, to ensure all-day and automatic self-monitoring of the activity behavior, the Active Coach app works in combination with a wearable activity tracker, the Fitbit Charge. The Fitbit Charge is a wrist-worn activity tracker that uses a 3-axis accelerometer to track a person’s movement [87]. The wristband can track the number of steps walked, active minutes, floors climbed, the quality of sleep, and other personal metrics. For the Active Coach app, only the number of steps walked was used. Fitbit trackers are valid and reliable devices for measuring step counts in healthy young adults [20,88].

The Active Coach app includes a registration process and 7 other components, each with their own influence on the determinants (Table 3). In the focus groups conducted in this study, participants said they were willing to go through a registration process at the beginning of an app to receive more personal information:

...the more personal an app, the better. You need to register for almost every site or app, I don’t mind. As long as it’s not a whole questionnaire.

However, participants indicated that the questions of the registration process should not be too detailed, too long, or go back too far in the past. Therefore, the registration process consists of only 3 screens. First, personal questions (name, email-address, password, gender, date of birth) are asked. Second, it is asked what kind of job the user has (mostly sitting or mostly standing or walking), and how the user would like to become more active (PA or AT). Third, perceived benefits are asked regarding the chosen behavior. If a user chooses PA, answer options are as follows: (1) being fit and healthy, (2) looking good (weight maintenance, appearance), and (3) relaxing, having distraction and/or social contact. If a user choses AT, answer options are as follows: (1) being fit and healthy, (2) saving money (no fuel costs), and (3) practical reasons (no traffic jams, not searching for parking spots).

**Figure 2:**
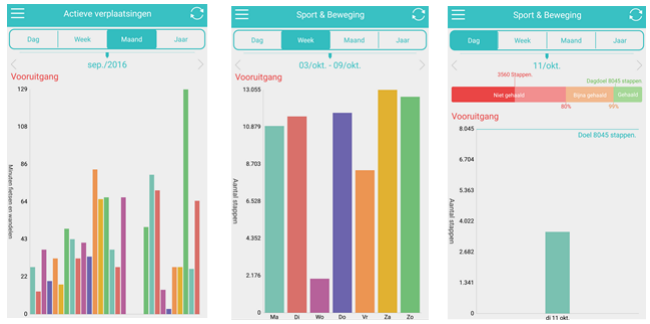
Active Coach app screenshots with (a) month overview of AT in min per day, (b) week overview of PA in steps per day, and (c) day overview of PA in steps PA per day and a personal goal line and bar.

After completing the registration, the Active Coach app consists of a 9-week program. During those 9 weeks, user’s PA behavior is being tracked by the Fitbit Charge (step count) and their AT behavior is being tracked by mobile smartphone sensors (GPS and accelerometer). Regardless of the activity choice (PA or AT), both behaviors will be tracked automatically and will be visible for the user in the app on a graphical display (steps/day [PA] and minutes/day [AT] per day, week, month, and year) in the app (Figure 2). However, the goal setting and the information received will differ according to the chosen behavior.

**Table 3:** The 8 components of the Active Coach app with their behavior change techniques (BCTs) and determinants.

Active Coach component	Behavior change techniques	Determinants
Registration process	N/A^a^	N/A
Tracking of PA^b^ (via Fitbit) and AT^c^ (via mobile sensors) + graphical display	Self-monitoring	Self-efficacy Knowledge
1 week baseline activity level measuring	Self-monitoring	Self-efficacy Knowledge
Weekly goal (steps/day or min AT^c^/day) (set by app)	Goal-setting	Self-efficacy
End of each week: feedback on goal achievement goal achieved: option to increase goal (user’s choice) goal not achieved: perceived barriers option to decrease goal (user’s choice)	Feedback on behavior Goal-setting Review behavior goals	Self-efficacy
	Prompts/cues	Self-efficacy (app use)
Daily visual feedback on goal achievement	Feedback on behavior	Self-efficacy
Practical tips: 2 per week	Instruction on how to perform the behavior	Self-efficacy Knowledge Attitude
	Enhancing network linkages	Social support
	Prompts/cues	Self-efficacy (app use)
Facts: 1 per week	Information about health consequences	Knowledge Attitude
	Enhancing network linkages	Social support
	Prompts/cues	Self-efficacy (app use)

^a^N/A: not applicable.

^b^PA: physical activity.

^c^AT: active transport.

The first week of the 9-week program is a baseline week during which the baseline activity level of the user is measured. At the end of this week, a personal goal dependent on the chosen behavior (PA in steps/day or AT in min/day) is set by the app for the following week (eg, Your goal for next week is to try and walk 6000 steps each day). The goal was based on mean steps per day or min AT per day of at least 3 of the 7 baseline days, which was increased by 10%. Participants of the focus groups conducted in this study agreed that there should be a goal or a target to reach for. Some wanted it to be set automatically, whereas others wanted to choose their own goal.

Therefore, the app provides both automatic and individually set goals. The focus group participants also liked receiving rewards (eg, virtual rewards such as a medal) or positive feedback when they achieve a goal or target. Therefore, every day during the following 8 weeks, users receive a notification on whether or not they achieved their day goal. They can also see their daily and weekly goal progression on the graphs in the app (Figure 2). In addition, users receive feedback on their goal achievement at the end of each week (Sunday). If they achieve their goal, they can increase it with 10%, 25%, or 50% or they can maintain the same goal for the next week. If they do not achieve their goal, they can choose to decrease it with 10%, 25%, or 50% or they can maintain the same goal for the next week. Additionally, users are asked why they did not achieve their goal (perceived barriers). For a user who chooses PA, answer options are as follows: (1) lack of time, motivation, energy, etc; (2) lack of money, sports equipment, etc; (3) no sports partner; and (4) bad health (sick, injury, tired). For a user who chooses AT, answer options were as follows: (1) feeling unsafe (dangerous traffic, bike theft), (2) practical reasons (bad weather, luggage), (3) laziness or habit of taking the car, and (4) bad health (sick, injury, tired). This information is being used to give users more personal feedback.

Every Monday and Friday during the 8 weeks after the baseline week, users receive a notification with a practical tip, and every Wednesday, they receive a notification with a scientific fact to help and motivate them to reach their goal. The content of the tips and facts is tailored based on information from the registration process (gender, sitting or standing job, PA or AT, selected benefits), on goal achievement, and on the selected barriers. If users achieve their goal from the previous week, they receive 1 general tip (eg, Naviki is a very practical app to help plan your route with information on slopes and the weather on the road) and fact (eg, Did you know that being active when you’re young decreases the risk of heart disease later in life?) and 1 tip adapted according to the benefits selected during the registration (eg, benefit: staying fit and healthy, tip: You can stay fit and healthy together with your colleagues by joining a fun biking contest). If users do not achieve their goal from the previous week, they receive 2 tips adapted according to the barriers selected at the end of the previous week (eg, barrier: high costs, tip: By asking your colleagues to make a walk during lunch break, you can be more active at work without any extra costs). Users can select the preferred time to receive these notifications. When pretesting the tips and facts with lower-educated working young adults, they indicated that some tips and facts were too long, too commanding, and too obvious. With their suggestions, tips and facts were adapted to be shorter, more powerful, and more supportive. New information was also added, and some written messages were replaced by figures.

### Step 4: Testing the App on Usability, Acceptability, and Feasibility

The volunteers (n=11) who tested the Active Coach app for technical errors and faults all owned different brands of Android smartphones. Several problems occurred such as too small fonts, errors in texts, not receiving messages, receiving wrong messages, receiving notifications at the wrong time, and error messages. All these problems were fixed. The main change was adding an extra page in the app to collect all received notifications about tips, facts, and goals, so that the users could consult these again at any moment.

Next, lower-educated working young adults (n=16) tested the app on acceptability and feasibility. Two participants dropped out due to a damaged smartphone. On the basis of the interviews, participants were very positive about the simplicity of the Active Coach app. They used and understood the app without any problems:

I think the app is easy and very clear. And there is not too much in it. With some apps you are like “Where do I find that again?”, but that is not a problem with this one.

Participants were also positive about the design of the app:

I think it is very clear, neat and with nice colors. Modern, also.

Participants had no problems with the registration process: they liked that it was not too long and they understood all the questions. Although they did not want more questions during the registration process, it was said that the app (particularly the tips) should be more tailored:

Some tips you can actually use, but other tips are not very applicable for you. Those are more general tips. It would be better if they were more personal.

However, they really liked the scientific facts and thought they were very interesting. In addition, participants also liked that they received the tips and facts as notifications, because it reminded them to have a look at the app. Participants indicated that they regularly looked at the notifications page to reread some tips and facts.

Participants were positive about the goals. They particularly liked the weekly possibility to increase or decrease their goal:

I really like the goals, it motivates you. And it is good that you can change your goal each week, with the 4 options.

They also liked the graphical display of the tracked behavior and the goals (“It is very clear, with all the different colors. And also divided in day, week, month, year. That’s good.”), although they had some suggestions for improvement:

You can only see your current goal. It would be practical if, when you look at the month, it would display multiple marks for the goals of each week.

In the beginning, participants complained about battery drainage, mostly because their Bluetooth, GPS, and/or Internet connection was switched on the entire day. However, they quickly fixed this problem themselves:

I always have my GPS switched on, but not my Bluetooth. I just switch it on once a day, in the evening, to sync everything. So now my battery use is under control.

Finally, participants really liked the Fitbit wearable:

You don’t even feel it, it doesn’t bother me at all. And it is very secure, it doesn’t come off easily.

However, they found it to be a disadvantage that it is not waterproof and they could not wear it while swimming. Some participants also looked at the native Fitbit app, mainly to check additional features such as calories burned or sleep.

Results from the questionnaire during the fourth and last interview showed that 11 of the 14 participants thought that they were more active because of the Active Coach app and also 11 of the 14 would recommend the app to others. Furthermore, as shown in Table 4, almost all participants (13 of 14) agreed or strongly agreed that the app was clear and understandable and that the tips and facts were understandable. In addition, more than half agreed or strongly agreed that it was motivating to have a goal and useful to receive weekly feedback about that goal. On the basis of these results, adaptations were made to the app. Some minor problems with the notifications page were fixed, and the list of practical tips was re-evaluated.

Results from Google Analytics showed that the Active Coach app was most used on days and hours that users received notifications with tips, facts, and weekly feedback on goal achievements (Monday, Wednesday, Friday, and Sunday between 8 and 10 PM). It also showed that they used the app on average 1 min per session. The app was visited by minimum 1 and maximum 14 users on each day of the acceptability and feasibility testing period with a minimum of 5 sessions a day and a maximum of 31 sessions a day.

**Table 4:** Experiences with the Active Coach app (results from the questionnaire at week 9). Response categories: 5-point scale, from 1 (strongly disagree) to 5 (strongly agree).

Active Coach app		Strongly disagree (n)	Disagree (n)	Sometimes (dis)agree (n)	Agree (n)	Strongly agree (n)	Mean (SD)
**What did you think about the Active Coach app?**				
	Clear		1		11	2	4.0 (0.7)
	Pretty		1	5	6	2	3.6 (0.8)
	Boring	1	5	5	2	1	2.8 (1.1)
	Understandable			1	8	5	4.3 (0.6)
	Fun		2		9	3	3.9 (0.9)
	Attractive		2	5	6	1	3.4 (0.9)
	User-friendly		1	2	8	3	3.9 (0.8)
**What did you think about the tips you received?**				
	Interesting		3	4	4	3	3.5 (1.1)
	Motivating		3	6	3	2	3.3 (0.9)
	Boring	2	5	4	3		2.6 (1.0)
	Useful	1	1	5	5	2	3.4 (1.1)
	Understandable		1		6	7	4.5 (0.8)
	Commanding	5	7		1	1	2.0 (1.2)
**What did you think about the facts you received?**				
	Interesting	1		3	7	3	3.8 (1.1)
	Motivating	1	2	5	5	1	3.2 (1.1)
	Boring	5	3	4	2		2.2 (1.1)
	Believable	1		1	7	5	4.1 (1.1)
	Understandable	1			4	9	4.4 (1.1)
	Educational	1	1	4	5	3	3.6 (1.1)
**What did you think about the goals you received?**				
	I tried to achieve my daily goal			7	6	1	3.6 (0.6)
	I found it motivating to have a goal			5	4	5	4.0 (0.9)
	I found it useful to receive daily feedback	1	4	2	6	1	3.1 (1.1)
	I found it useful to receive weekly feedback	1		1	8	4	4.0 (1.0)
	I liked it that I could adjust my goal weekly	1	1	1	6	5	3.9 (1.2)

## Discussion

This study aimed to describe all steps of the development (including the usability, acceptability, and feasibility testing) of a new smartphone app (Active Coach) that will be used, in combination with a wearable activity tracker, in an intervention to promote an active lifestyle in lower educated working young adults. It is important to use a stepwise and iterative approach when developing new smartphone apps. The literature in this area clearly emphasizes the importance of formative research and pretesting and indicates they are necessary steps before conducting a pilot test or RCT [34]. In this study, exploratory focus groups during the formative research revealed characteristics of the target group that were not known beforehand. For example, wrist-worn activity trackers were only included after statements of focus group participants that they were not allowed to carry their smartphone with them during working hours. Lower-educated working young adults often have blue-collar jobs during which it is prohibited to carry a smartphone, thus relying on the built-in sensors of smartphones to track activity would have resulted in incomplete and incorrect data. An Australian study on interest and preferences for using activity tracking devices [89] showed that activity trackers should indeed align with the characteristics of a target group. They found that accelerometers (eg, Fitbit) are preferred, especially among younger people, because of their wearing position (ie, wrist), features (ie, measures steps), and characteristics (ie, accuracy) [89].

The use of the Fitbit Charge wearable activity tracker was positively evaluated in the acceptability and feasibility test of this study. Cadmus-Bertram et al [90,91] also found low barriers and very high adherence regarding Fitbit use in an RCT among obese, middle-aged women. Lower-educated working young adults in this study found the Fitbit Charge easy and comfortable to wear and use, and they liked the long battery life. It automatically tracked their activity behavior all day, even during working hours and during (sport) activities when they did not carry their phone with them. A disadvantage was that it is not waterproof [87], which means that swimming cannot be tracked. However, this issue might be resolved soon as future generations of activity trackers will probably be waterproof. Next, because of the relatively high cost, lower-educated working young adults might not own a Fitbit tracker or are not willing to purchase it in the future. Nevertheless, a study among Australian adults found that cost is not a significant barrier to the use of activity trackers [89]. Additionally, it is expected that prices of wearable activity trackers will drop quickly in the future, as this technology continues to evolve rapidly [92].

Focus group participants also indicated that they prefer information in apps to be personal. Previously, it has been shown that individually tailored feedback and advice (ie, based on the user’s own characteristics [93]) is more likely to be effective than generic information about PA [94-96]. The use of the wearable activity tracker allowed the Active Coach app to provide personal activity information (eg, graphs, goals), without manual user input, which is a great strength of the developed app [67,97]. It has been shown that that user engagement in health behavior programs is much better when automatic tracking is applied [97]. Providing tailored advice (personal tips and facts) remains more difficult, as it requires knowledge about people’s characteristics. In computer-tailored interventions, tailored advice is based on participants’ answers to a predefined diagnostic questionnaire. However, a recent US study on health app use found that the primary reason respondents stopped using health-related apps was the demanding nature of manual data entry [98]. In this study, focus group participants said that, although they were willing to complete a registration process to receive more personal information, questions should be limited in number and should not be too long or too detailed. Therefore, the provided advice in the Active Coach app (tips and facts) is only tailored to a certain extent, by using information from the concise registration process, the weekly goal achievements, and the reported barriers. Results from the acceptability and feasibility test showed that participants had no problems with the registration questions, but they thought the tips needed to be more personal. This indicates the difficult balance between manual data entry burden and providing app users with tailored advice. A possible solution would be to provide tailored advice based on automatically gathered information. Klein et al [67] described the use of location data (GPS) to monitor user’s actual location and to identify frequently visited locations to send timely and context-specific messages. A very advanced example of this is Google Now, which is an intelligent virtual assistant app that learns from user’s behaviors, habits, and preferences (based on location data, weather information, Internet search history, online agenda, email data, etc) and shows relevant information without the user asking for it [99]. This information might also cover other health behaviors besides PA, such as healthy nutrition or sleep quality, depending on the interests of the user. The feasibility and acceptability test showed indeed that some lower-educated working young adults expressed interest in other health features of the Fitbit app, such as burned calories or sleep. However, this type of automatically generated tailored information would require expert technological knowledge and skills, a lot of time, and extensive financial means to add this to the existing app. Although the Active Coach app was developed in cooperation with a commercial mobile app development company, the available time and limited financial means only allowed for concise tailoring of advice. Future mHealth interventions should keep the importance of both tailored health information and tailored advice in mind while attempting to limit data entry burden as much as possible.

A limitation of this study is that some app features could not be realized because of limited time and financial means, regardless of the target group’s interest in it, such as highly personalized advice and virtual rewards for goal achievement. Future mHealth studies might want to include these elements. Next, the choice to develop a native Android app ensures the best user experience and compatibility with smartphone’s native features and hardware [71]. However, this means that people with iPhone (IOS) or Windows phone cannot use the Active Coach app. The app is specifically adapted to the Flemish lower-educated working young adults, which limits its generalizability. However, targeting apps to specific population groups may also enhance their efficacy [18]. Furthermore, it is possible to adjust elements of the app (eg, translate it to other languages, adapt the tips and facts) to make it useable for other target groups.

This study includes some important strengths. The stepwise development process, during which lower-educated working young adults were regularly consulted, resulted in a new PA app that is adapted to the needs and preferences of an under-researched target group at high risk for physical inactivity. The app was specifically adapted for the Flemish lower-educated working young adults on several levels such as language (eg, simple and understandable), layout (eg, tested by the target group to ensure user-friendliness, attractiveness), content (eg, tips on benefits of AT or PA focused on benefits that were important for the target group), and their lifestyle (eg, use of wearable activity trackers because the target group was not allowed to carry their smartphone with them during working hours). To the best of our knowledge, this is the first app aiming to promote an active lifestyle that is specifically developed for lower-educated working young adults. Several qualitative and quantitative research methods (focus group discussions, think-aloud interviews, semi-structured interviews, questionnaires, Google Analytics) were used to test and adapt multiple versions of the Active Coach app. As a result, many technical errors and faults could be eliminated to maximize the user-friendliness of the app. Furthermore, by identifying the theoretical constructs that needed to be targeted, integrating them into the ASE-model, and by using multiple self-regulatory BCTs, a theory- and evidence-based app was developed, which is important to increase effectiveness [37,96]. Finally, the use of a Fitbit wearable activity tracker allowed the Active Coach app to provide personal activity information (eg, graphs, goals), without manual user data input. As recommended in previous research [33], it is important to present the process of developing a new health app to help others in developing effective tools to improve health. The next step in this process is to test the efficacy of the Active Coach app in an RCT.

Research showed that formative research and pretesting before conducting a pilot test or RCT ensures the best chance of developing new and effective tools to improve health. Therefore, we used a stepwise and iterative approach during which the target group was regularly consulted to develop an evidence- and theory-based smartphone app promoting an active lifestyle that is adapted to the specific needs and preferences of lower-educated working young adults. At the end of the development process, the Active Coach app was overall positively evaluated by the target group.

## Abbreviations

AT: active transport
ASE: attitude–social influence–self-efficacy
BCTs: behavior change techniques
PA: physical activity

## References

[1] Arnett JJ (2000). Emerging adulthood - a theory of development from the late teens through the twenties. Am Psychol.

[2] Bell S, Lee C (2005). Emerging adulthood and patterns of physical activity among young Australian women. Int J Behav Med.

[3] Allender S, Hutchinson L, Foster C (2008). Life-change events and participation in physical activity: a systematic review. Health Promot Int.

[4] Dowda M, Ainsworth BE, Addy CL, Saunders R, Riner W (2003). Correlates of physical activity among US young adults, 18 to 30 years of age, from NHANES III. Ann Behav Med.

[5] Kwan MY, Cairney J, Faulkner GE, Pullenayegum EE (2012). Physical activity and other health-risk behaviors during the transition into early adulthood. Am J Prev Med.

[6] Departement Mobiliteit en Openbare Werken (2011). Mobielvlaanderen.

[7] Beige S, Axhausen KW (2012). Interdependencies between turning points in life and long-term mobility decisions. Transportation.

[8] Telama R (2009). Tracking of physical activity from childhood to adulthood: a review. Obes Facts.

[9] Drieskens S (2014). Scientific Institute of Public Health.

[10] Lee IM, Shiroma EJ, Lobelo F, Puska P, Blair SN, Katzmarzyk PT, Lancet Physical Activity Series Working Group (2012). Effect of physical inactivity on major non-communicable diseases worldwide: an analysis of burden of disease and life expectancy. Lancet.

[11] Sisson S, Tudor-Locke C (2008). Comparison of cyclists' and motorists' utilitarian physical activity at an urban university. Prev Med.

[12] He XZ, Baker DW (2005). Differences in leisure-time, household, and work-related physical activity by race, ethnicity, and education. J Gen Intern Med.

[13] Cerin E, Leslie E, Owen N (2009). Explaining socio-economic status differences in walking for transport: an ecological analysis of individual, social and environmental factors. Soc Sci Med.

[14] Scheepers E, Wendel-Vos W, van Kempen E, Panis LI, Maas J, Stipdonk H, Moerman M, den Hertog F, Staatsen B, van Wesemael P, Schuit J (2013). Personal and environmental characteristics associated with choice of active transport modes versus car use for different trip purposes of trips up to 7.5 kilometers in The Netherlands. PLoS One.

[15] Roskam AJ, Kunst Ae, Van Oyen H, Demarest S, Klumbiene J, Regidor E, Helmert U, Jusot F, Dzurova D, Mackenbach JP, for additional participants to the study (2010). Comparative appraisal of educational inequalities in overweight and obesity among adults in 19 European countries. Int J Epidemiol.

[16] Fanning J, Mullen S, McAuley E (2012). Increasing physical activity with mobile devices: a meta-analysis. J Med Internet Res.

[17] Monroe CM, Thompson DL, Bassett DR, Fitzhugh EC, Raynor HA (2015). Usability of mobile phones in physical activity–related research: a systematic review. Am J Health Educ.

[18] Schoeppe S, Alley S, Van Lippevelde W, Bray NA, Williams SL, Duncan MJ, Vandelanotte C (2016). Efficacy of interventions that use apps to improve diet, physical activity and sedentary behaviour: a systematic review. Int J Behav Nutr Phys Act.

[19] Dute DJ, Bemelmans WJ, Breda J (2016). Using mobile apps to promote a healthy lifestyle among adolescents and students: a review of the theoretical basis and lessons learned. JMIR Mhealth Uhealth.

[20] Evenson KR, Goto MM, Furberg RD (2015). Systematic review of the validity and reliability of consumer-wearable activity trackers. Int J Behav Nutr Phys Act.

[21] Riley WT, Rivera DE, Atienza AA, Nilsen W, Allison SM, Mermelstein R (2011). Health behavior models in the age of mobile interventions: are our theories up to the task?. Transl Behav Med.

[22] Smith A (2015). Pew Research Center.

[23] (2016). Advertising.sanoma.

[24] Rice RE, Katz JE (2003). Comparing internet and mobile phone usage: digital divides of usage, adoption, and dropouts. Telecomm Policy.

[25] Dennison L, Morrison L, Conway G, Yardley L (2013). Opportunities and challenges for smartphone applications in supporting health behavior change: qualitative study. J Med Internet Res.

[26] Buhi ER, Trudnak TE, Martinasek MP, Oberne AB, Fuhrmann HJ, McDermott RJ (2012). Mobile phone-based behavioural interventions for health: a systematic review. Health Educ J.

[27] Direito A, Dale LP, Shields E, Dobson R, Whittaker R, Maddison R (2014). Do physical activity and dietary smartphone applications incorporate evidence-based behaviour change techniques?. BMC Public Health.

[28] Conroy DE, Yang C, Maher JP (2014). Behavior change techniques in top-ranked mobile apps for physical activity. Am J Prev Med.

[29] Middelweerd A, Mollee JS, van der Wal CN, Brug J, Te Velde SJ (2014). Apps to promote physical activity among adults: a review and content analysis. Int J Behav Nutr Phys Act.

[30] West JH, Hall PC, Hanson CL, Barnes MD, Giraud-Carrier C, Barrett J (2012). There's an app for that: content analysis of paid health and fitness apps. J Med Internet Res.

[31] Cowan LT, Van Wagenen SA, Brown BA, Hedin RJ, Seino-Stephan Y, Hall PC, West JH (2013). Apps of steel: are exercise apps providing consumers with realistic expectations?: a content analysis of exercise apps for presence of behavior change theory. Health Educ Behav.

[32] Schoeppe S, Alley S, Rebar AL, Hayman M, Bray NA, Van Lippevelde W, Gnam JP, Bachert P, Direito A, Vandelanotte C (2017). Apps to improve diet, physical activity and sedentary behaviour in children and adolescents: a review of quality, features and behaviour change techniques. Int J Behav Nutr Phys Act.

[33] Kirwan M, Duncan MJ, Vandelanotte C, Mummery WK (2013). Design, development, and formative evaluation of a smartphone application for recording and monitoring physical activity levels: the 10,000 Steps “iStepLog”. Health Educ Behav.

[34] Whittaker R, Merry S, Dorey E, Maddison R (2012). A development and evaluation process for mHealth interventions: examples from New Zealand. J Health Commun.

[35] Yardley L, Morrison L, Bradbury K, Muller I (2015). The person-based approach to intervention development: application to digital health-related behavior change interventions. J Med Internet Res.

[36] Craig P, Dieppe P, Macintyre S, Michie S, Nazareth I, Petticrew M, Medical Research Council Guidance (2008). Developing and evaluating complex interventions: the new Medical Research Council guidance. Br Med J.

[37] Webb TL, Joseph J, Yardley L, Michie S (2010). Using the internet to promote health behavior change: a systematic review and meta-analysis of the impact of theoretical basis, use of behavior change techniques, and mode of delivery on efficacy. J Med Internet Res.

[38] Jaspers MW (2009). A comparison of usability methods for testing interactive health technologies: methodological aspects and empirical evidence. Int J Med Inform.

[39] Bartholomew L, Parcel G, Kok G, Gottlieb N, Fernandez M (2011). Planning Health Promotion Programs: An Intervention Mapping Approach.

[40] Michie S, Richardson M, Johnston M, Abraham C, Francis J, Hardeman W, Eccles MP, Cane J, Wood CE (2013). The behavior change technique taxonomy (v1) of 93 hierarchically clustered techniques: building an international consensus for the reporting of behavior change interventions. Ann Behav Med.

[41] Trost S, Owen N, Bauman A, Sallis J, Brown W (2002). Correlates of adults' participation in physical activity: review and update. Med Sci Sport Exer.

[42] Bauman AE, Reis RS, Sallis JF, Wells JC, Loos RJF, Martin BW, Lancet Physical Activity Series Working Group (2012). Correlates of physical activity: why are some people physically active and others not?. Lancet.

[43] Short CE, Vandelanotte C, Rebar A, Duncan MJ (2014). A comparison of correlates associated with adult physical activity behavior in major cities and regional settings. Health Psychol.

[44] de Geus B, De Bourdeaudhuij I, Jannes C, Meeusen R (2008). Psychosocial and environmental factors associated with cycling for transport among a working population. Health Educ Res.

[45] Verhoeven H, Simons D, Van Dyck D, Van Cauwenberg J, Clarys P, De Bourdeaudhuij I, de Geus B, Vandelanotte C, Deforche B (2016). Psychosocial and environmental correlates of walking, cycling, public transport and passive transport to various destinations in Flemish older adolescents. PLoS One.

[46] Panter JR, Jones A (2010). Attitudes and the environment as determinants of active travel in adults: what do and don't we know?. J Phys Act Health.

[47] Simons D, Clarys P, De Bourdeaudhuij I, de Geus B, Vandelanotte C, Deforche B (2014). Why do young adults choose different transport modes? A focus group study. Transp Policy.

[48] Titze S, Stronegger WJ, Janschitz S, Oja P (2007). Environmental, social, and personal correlates of cycling for transportation in a student population. J Phys Act Health.

[49] Shannon T, Giles-Corti B, Pikora T, Bulsara M, Shilton T, Bull F (2006). Active commuting in a university setting: Assessing commuting habits and potential for modal change. Transp Policy.

[50] Chinn DJ, White M, Harland J, Drinkwater C, Raybould S (1999). Barriers to physical activity and socioeconomic position: implications for health promotion. J Epidemiol Community Health.

[51] Salmon J, Owen N, Crawford D, Bauman A, Sallis JF (2003). Physical activity and sedentary behavior: a population-based study of barriers, enjoyment, and preference. Health Psychol.

[52] Myers RS, Roth DL (1997). Perceived benefits of and barriers to exercise and stage of exercise adoption in young adults. Health Psychol.

[53] Zunft HJ, Friebe D, Seppelt B, Widhalm K, Remaut-De Winter AM, Vaz de Almeida MD, Kearney JM, Gibney M (1999). Perceived benefits and barriers to physical activity in a nationally representative sample in the European Union. Public Health Nutr.

[54] Hunter R, Tully M, Donnelly P, Stevenson M, Kee F (2014). Knowledge of UK physical activity guidelines: implications for better targeted health promotion. Prev Med.

[55] Knox EC, Musson H, Adams EJ (2015). Knowledge of physical activity recommendations in adults employed in England: associations with individual and workplace-related predictors. Int J Behav Nutr Phys Act.

[56] World Health Organization, Regional Office for the Eastern Mediterranean (2012). Health education: theoretical concepts, effective strategies and core competencies: a foundation document to guide capacity development of health educators.

[57] Wendel-Vos W, Droomers M, Kremers S, Brug J, van Lenthe F (2007). Potential environmental determinants of physical activity in adults: a systematic review. Obes Rev.

[58] Tansey O (2007). Process tracing and elite interviewing: a case for non-probability sampling. PS Polit Sci Polit.

[59] Trotter 2nd RT (2012). Qualitative research sample design and sample size: resolving and unresolved issues and inferential imperatives. Prev Med.

[60] Krueger R, Morgan D (1994). Developing questions for focus groups: Focus group kit 3.

[61] Strauss A (1987). Qualitative analysis for social scientists.

[62] Michie S, Johnston M (2013). Behavior Change Techniques. Encyclopedia of Behavioral Medicine.

[63] Williams SL, French DP (2011). What are the most effective intervention techniques for changing physical activity self-efficacy and physical activity behaviour--and are they the same?. Health Educ Res.

[64] Michie S, Abraham C, Whittington C, McAteer J, Gupta S (2009). Effective techniques in healthy eating and physical activity interventions: a meta-regression. Health Psychol.

[65] Greaves CJ, Sheppard KE, Abraham C, Hardeman W, Roden M, Evans PH, Schwarz P, IMAGE Study Group (2011). Systematic review of reviews of intervention components associated with increased effectiveness in dietary and physical activity interventions. BMC Public Health.

[66] Conn VS, Hafdahl AR, Mehr DR (2011). Interventions to increase physical activity among healthy adults: meta-analysis of outcomes. Am J Public Health.

[67] Klein M, Manzoor A, Middelweerd A, Mollee J, te Velde S (2015). Encouraging physical activity via a personalized mobile system. Ieee Internet Comput.

[68] Cremers H, Mercken L, Crutzen R, Willems P, de Vries H, Oenema A (2014). Do email and mobile phone prompts stimulate primary school children to reuse an Internet-delivered smoking prevention intervention?. J Med Internet Res.

[69] Crutzen R, de Nooijer J, Brouwer W, Oenema A, Brug J, de Vries NK (2011). Strategies to facilitate exposure to internet-delivered health behavior change interventions aimed at adolescents or young adults: a systematic review. Health Educ Behav.

[70] Svensson M, Svensson T, Hansen AW, Trolle Lagerros Y (2012). The effect of reminders in a web-based intervention study. Eur J Epidemiol.

[71] Charland A, Leroux B (2011). Mobile application development: web vs. native. Commun ACM.

[72] (2015). International Data Corporation.

[73] Vansteenkiste M, Simons J, Lens W, Soenens B, Matos L (2005). Examining the motivational impact of intrinsic versus extrinsic goal framing and autonomy-supportive versus internally controlling communication style on early adolescents' academic achievement. Child Dev.

[74] Ryan RM, Deci EL (2000). Self-determination theory and the facilitation of intrinsic motivation, social development, and well-being. Am Psychol.

[75] Nielsen J (2000). NNGroup.

[76] Van den Haak MJ, De Jong MD, Schellens PJ (2007). Evaluation of an informational web site: three variants of the think-aloud method compared. Tech Commun.

[77] De Cocker K, Spittaels H, Cardon G, De Bourdeaudhuij I, Vandelanotte C (2012). Web-based, computer-tailored, pedometer-based physical activity advice: development, dissemination through general practice, acceptability, and preliminary efficacy in a randomized controlled trial. J Med Internet Res.

[78] De Cocker K, De Bourdeaudhuij I, Cardon G, Vandelanotte C (2015). Theory-driven, web-based, computer-tailored advice to reduce and interrupt sitting at work: development, feasibility and acceptability testing among employees. BMC Public Health.

[79] Crutzen R, Roosjen JL, Poelman J (2012). Using Google Analytics as a process evaluation method for Internet-delivered interventions: an example on sexual health. Health Promot Int.

[80] De Vries H, Backbier E, Kok G, Dijkstra M (1995). The impact of social influences in the context of attitude, self-efficacy, intention, and previous behavior as predictors of smoking onset. J Appl Soc Psychol.

[81] Vonk Noordegraaf A, Huirne JA, Pittens CA, van Mechelen W, Broerse JE, Brölmann HA, Anema JR (2012). eHealth program to empower patients in returning to normal activities and work after gynecological surgery: intervention mapping as a useful method for development. J Med Internet Res.

[82] van Oostrom SH, Anema JR, Terluin B, Venema A, de Vet Henrica CW, van Mechelen W (2007). Development of a workplace intervention for sick-listed employees with stress-related mental disorders: Intervention Mapping as a useful tool. BMC Health Serv Res.

[83] Vermeulen SJ, Anema JR, Schellart AJ, van Mechelen w, van der Beek AJ (2009). Intervention mapping for development of a participatory return-to-work intervention for temporary agency workers and unemployed workers sick-listed due to musculoskeletal disorders. BMC Public Health.

[84] Noar SM, Crosby R, Benac C, Snow G, Troutman A (2009). Application of the attitude-social influence-efficacy model to condom use among African-American STD clinic patients: implications for tailored health communication. AIDS Behav.

[85] Belmon LS, Middelweerd A, Te Velde Saskia J, Brug J (2015). Dutch young adults ratings of behavior change techniques applied in mobile phone apps to promote physical activity: a cross-sectional survey. JMIR Mhealth Uhealth.

[86] Guertler D, Vandelanotte C, Kirwan M, Duncan MJ (2015). Engagement and nonusage attrition with a free physical activity promotion program: the case of 10,000 steps Australia. J Med Internet Res.

[87] (2016). Fitbit.

[88] Takacs J, Pollock CL, Guenther JR, Bahar M, Napier C, Hunt MA (2014). Validation of the Fitbit One activity monitor device during treadmill walking. J Sci Med Sport.

[89] Alley S, Schoeppe S, Guertler D, Jennings C, Duncan M, Vandelanotte C (2016). Interest and preferences for using advanced physical activity tracking devices: results of a national cross-sectional survey. BMJ Open.

[90] Cadmus-Bertram L, Marcus BH, Patterson RE, Parker BA, Morey BL (2015). Use of the Fitbit to measure adherence to a physical activity intervention among overweight or obese, postmenopausal women: self-monitoring trajectory during 16 weeks. JMIR Mhealth Uhealth.

[91] Cadmus-Bertram LA, Marcus BH, Patterson RE, Parker BA, Morey BL (2015). Randomized trial of a Fitbit-based physical activity intervention for women. Am J Prev Med.

[92] PriceSpy.

[93] Kreuter MW, Strecher VJ, Glassman B (1999). One size does not fit all: The case for tailoring print materials. Ann Behav Med.

[94] Lustria ML, Noar SM, Cortese J, Van Stee SK, Glueckauf RL, Lee J (2013). A meta-analysis of web-delivered tailored health behavior change interventions. J Health Commun.

[95] Noar SM, Benac CN, Harris MS (2007). Does tailoring matter? Meta-analytic review of tailored print health behavior change interventions. Psychol Bull.

[96] Foster C, Richards J, Thorogood M, Hillsdon M (2013). Remote and web 2.0 interventions for promoting physical activity. Cochrane Database Syst Rev.

[97] Kim JY, Wineinger NE, Taitel M, Radin JM, Akinbosoye O, Jiang J, Nikzad N, Orr G, Topol E, Steinhubl S (2016). Self-monitoring utilization patterns among individuals in an incentivized program for healthy behaviors. J Med Internet Res.

[98] Krebs P, Duncan DT (2015). Health app use among US mobile phone owners: a national survey. JMIR Mhealth Uhealth.

[99] (2016). Digitaltrends.

